# HpaXpm, a novel harpin of *Xanthomonas phaseoli* pv. *manihotis*, acts as an elicitor with high thermal stability, reduces disease, and promotes plant growth

**DOI:** 10.1186/s12866-019-1691-4

**Published:** 2020-01-06

**Authors:** Yue Liu, Xiaoyun Zhou, Wenbo Liu, Jiamin Huang, Qinghuan Liu, Jianzhang Sun, Xinfeng Cai, Weiguo Miao

**Affiliations:** 1 Institute of Tropical Agriculture and Forestry, Hainan University, Haikou, Hainan Province China; 2 Key Laboratory of Green Prevention and Control of Tropical Plant Diseases and Pests (Hainan University), Ministry of Education, Haikou, Hainan Province China

**Keywords:** *Xanthomonas*, Harpin, Plant disease, Protein structure, Classification, Hypersensitive response, Defense response, Plant growth, Thermal stability

## Abstract

**Background:**

Harpins are proteins secreted by the type III secretion system of Gram-negative bacteria during pathogen–plant interactions that can act as elicitors, stimulating defense and plant growth in many types of non-host plants. Harpin-treated plants have higher resistance, quality and yields and, therefore, harpin proteins may potentially have many valuable agricultural applications. Harpins are characterized by high thermal stability at 100 °C. However, it is unknown whether harpins are still active at temperatures above 100 °C or whether different temperatures affect the activity of the harpin protein in different ways. The mechanism responsible for the heat stability of harpins is also unknown.

**Results:**

We identified a novel harpin, HpaXpm, from the cassava blight bacteria *Xanthomonas phaseoli* pv*. manihotis* HNHK. The predicted secondary structure and 3-D structure indicated that the HpaXpm protein has two β-strand domains and two major α-helical domains located at the N- and C-terminal regions, respectively. A phylogenetic tree generated using the maximum likelihood method grouped HpaXpm in clade I of the Hpa1 group along with harpins produced by other *Xanthomonas* spp. (i.e., HpaG-Xag, HpaG-Xcm, Hpa1-Xac, and Hpa1Xm). Phenotypic assays showed that HpaXpm induced the hypersensitive response (HR), defense responses, and growth promotion in non-host plants more effectively than Hp1Xoo (*X. oryzae* pv. *oryzae*). Quantitative real-time PCR analysis indicated that HpaXpm proteins subjected to heat treatments at 100 °C, 150 °C, or 200 °C were still able to stimulate the expression of function-related genes (i.e., the HR marker genes *Hin1* and *Hsr203J*, the defense-related gene *NPR1*, and the plant growth enhancement-related gene *NtEXP6*); however, the ability of heat-treated HpaXpm to induce HR was different at different temperatures.

**Conclusions:**

These findings add a new member to the harpin family. HpaXpm is heat-stable up to 200 °C and is able to stimulate powerful beneficial biological functions that could potentially be more valuable for agricultural applications than those stimulated by Hpa1Xoo. We hypothesize that the extreme heat resistance of HpaXpm is because the structure of harpin is very stable and, therefore, the HpaXpm structure is less affected by temperature.

## Background

Harpins, encoded by *hrp* (hypersensitive response and pathogenicity) genes of Gram-negative bacteria, are secreted by the type III secretion system during pathogen–plant interactions [1–5]. Based on homologous regions in *Xanthomonas* species, the *hrp* cluster contains *hrp*, *hrc* (*hrp*-conserved), and *hpa* (*hrp*-associated) genes [5–7]. Among these genes, the *hpa* gene plays a supporting role in inducing host pathogenic or non-host disease resistance. Strains with *hpa* gene mutations generally do not exhibit phenotypic changes in disease symptoms of the same severity as those with *hrp* or *hrc* gene mutations [6, 8, 9].

To date, multiple harpins have been identified [4, 9–13]. In a recent review [2], harpins were categorized in the following five major groups based on protein similarity and domain structures: the HrpN group, the HrpZ1 group, the HrpW1 group, the Hpa1 group, and an ‘Others’ group, which includes some unclassified harpins. Moreover, it has been suggested that the Hpa1 group is divided into two subgroups [3], with one subgroup containing the HpaG-Xag protein of *X. axonopodis* pv. *glycines*, Hpa1Xac of *X. axonopodis* pv. *citri*, and Hpa1-Xm of *X. citri* subsp. *malvacearum*, and the other subgroup containing the Hpa1Xoo protein of *X. oryzae* pv. *oryzae* and Hpa1Xoc of *X. oryzae* pv. *oryzicola* [3]. Harpins belonging to the Hpa1 group have been derived from pathogens of citrus [14], soybean [15], rice [16, 17], pepper [11], and cotton [10] crops. To date, there have been no reports of harpins derived from cassava pathogens. Cassava (*Manihot esculenta* Crantz) is a particularly important cash crop [18, 19] in the tropics, where it is considered a staple crop and one of the main sources of calories for more than one billion people [18, 20]. The most important bacterial disease of cassava is cassava bacterial blight, which is caused by the Gram-negative bacterium *Xanthomonas phaseoli* pv*. manihotis* (*Xpm*) [18, 20]. The characterization of new harpin members improves our understanding of the evolutionary relationships of harpins and provides more possibilities for understanding the mechanism of harpins that underlies the interaction between pathogens and plants.

Harpins share several common characteristics: they are glycine-rich (but lack cysteine), acidic, and have high thermal stability [1, 2]. To date, most research studies on harpins have focused on their biological activities [13, 21, 22]. For example, many studies have characterized harpin functional domains [3, 12, 23, 24] and the roles of harpins in non-host plants [25–27]. Only a few research studies have assessed the thermal stability of harpins. Investigations of harpin heat resistance have generally been carried out using harpin that has been boiled for 10 min [3, 10]. However, the maximum temperature that harpin protein can withstand is unknown; whether different temperatures affect the activity of harpin protein in different ways is unclear; and whether the high thermal stability of the harpin protein is due to structural specificity or because the structure has no effect on the thermal stability of harpin is also unknown.

Harpins can function as effectors to trigger hypersensitive response (HR) activity, establish systemic-acquired resistance (SAR), and confer plants with diverse beneficial effects such as quality and yield improvements. For instance, HrpN of *Erwinia amylovora* [28] was reported in 1992 as a cell-free elicitor of the HR and can induce disease resistance through the SAR pathway in non-host *Arabidopsis* plants [29]. HrpN can also activate abscisic acid signaling to induce drought tolerance in *Arabidopsis* [30]. The HrpZ protein of *Pseudomonas syringae* pv. *phaseolicola* enhances resistance to rhizomania disease in transgenic *Nicotiana benthamiana* and sugar beet [31]. The Hpa1-Xag protein of *Xanthomonas axonopodis* pv. *glycines* can elicit a typical HR in tobacco [14]. The HpaG-Xooc protein of *X. oryzae* pv. *oryzicola* can elicit a HR, which can induce disease- and insect-resistance in plants, and can promote plant growth [13]. The fragment Hpa1-Xm35–51 of *X. citri* subsp. *malvacearum* or the fragment Hpa1Xoo36–52 of *X. oryzae* pv. *oryzae* (*Xoo*) are sufficient to induce the HR [3]. The fragment Hpa1-Xm10–39 of *Xm* or Hpa1Xoo10–40 of *Xoo* can promote plant growth [3]. Furthermore, harpins can activate ethylene signaling to confer the plant with resistance to attacks by insects and stimulate plant growth [24]. In summary, harpins can stimulate plants to produce a variety of beneficial properties. However, to enhance the levels of resistance, quality and yield conferred to plants by harpin treatments, further investigations are needed to identify new harpin proteins and to screen for harpins that are likely to be the most valuable for agricultural applications.

In this study, we describe a new member of the harpin family, HpaG-Xpm (HpaXpm), and add to our understanding of the evolutionary relationships between harpins from *Xanthomonas* spp. We also subjected HpaXpm to different degrees of heat treatment to investigate whether HpaXpm is still active at 150 °C or 200 °C and to determine whether there are any differences in HpaXpm-excited HR activity after treatment at different temperatures. These investigations lay a theoretical foundation for exploring the heat-resistance mechanism of this protein in future studies. Furthermore, we compared HpaXpm and Hpa1Xoo activity when applied as a plant treatment to evaluate their ability to stimulate HR, defense responses, and plant growth to ascertain their potential use in agricultural applications.

## Methods

### Expression, purification, and western blot analysis of HpaXpm

*Xanthomonas phaseoli* pv. *manihotis* HNHK (*Xpm*) was identified by the Key Laboratory of Green Prevention and Control of Tropical Plant Diseases and Pests (Hainan University), Ministry of Education, Haikou, Hainan Province, China. The strains *Xpm* and BL21/pGEX-EF, were maintained in glycerol in the laboratory at − 80 °C. The *Xpm* strain was cultured in NA medium [32] at 28 °C. *Escherichia coli* BL21 (DE3) was cultured in LB medium with a final concentration of 100 μg ml^− 1^ ampicillin at 37 °C [10].

PCR was used to clone the entire *HpaXpm* gene from *Xpm* genomic DNA. The primers HpaXpm-F (5′-GGATCCAGTTAATCAGAGAGGAATCGTCATG-3′) and HpaXpm-R (5′-GAGCTCGGTAGGGGCGACCAACAGTTCGTTA-3′) were designed based on the *HpaXpm* sequence (GenBank accession No. KY765410.1). The *HpaXpm* sequence was inserted into the pGEX-EF vector, which allowed fusion with GST-tag-encoding nucleotides extracted from BL21/pGEX-EF that had been previously digested with the restriction enzymes *BamH1* and *Sac1*. The recombinant plasmid pGEX-GSTHpaXpm was transformed into *Escherichia coli* BL21 (DE3) (TransGen Biotech, Beijing, China). Next, the GST-HpaXpm were prepared according to previous methods [10]. The recombinant cells BL21/pGEX-GSTHpaXpm were grown in liquid LB medium up to 0.8 at OD_600_ nm at 37 °C. In order to explore conditions that can effectively induce overexpression of the GST-HpaXpm, isopropyl-β-D-thiogalactopyranoside (IPTG) was added to a final concentration of 0.05 mM or 0.1 mM for 3 h (h) or 5 h at 28 °C or 37 °C, respectively. To overproduce GST-HpaXpm, each of these variables was combined. The bacterial cells were harvested by centrifugation, suspended in phosphatebuffered saline (PBS), and then broken by ultrasonic treatment as previously described [10]. After centrifugation at 3,500 *g* for 5 min (min), the soluble (crude protein) and insoluble proteins were respectively gathered and identified by performing 12% SDS-PAGE. As previously described [33], the GST-HpaXpm was purified from crude protein using a GST-tag protein purification kit (Beyotime, Shanghai, China), and then digested by thrombin (GE, Boston, MA, USA) at 22 °C for 16 h. Western blot analysis was conducted to examine the expression efficiency and assembly of GST-HpaXpm, which were detected by performing SDS-PAGE and then transferred onto a polyvinylidene fluoride membrane. The membrane was blotted with a polyclonal antibody developed against GST and a goat anti-rabbit lgG-HRP antibody.

### Sequence alignment, characteristics analysis, structural prediction, and phylogenetic relationship

The *Xpm* genome sequence (taxid: 1985254) has been released by NCBI. Nucleotide sequences of *Xanthomonas hpa1*, including *hpa1Xac* (AAM35307.1), *hpa1Xag* (AF499777.1), *hpa1-Xm* (DQ643828.1), *hpa1Xoo* (AP008229.1), and *hpa1Xoc* (CP011957.1) were compared with the genome sequence of *Xpm*. Nucleotide and amino acid sequence alignments were analyzed using NCBI Blast (https://blast.ncbi.nlm.nih.gov/Blast.cgi). The basic characteristics of HpaXpm were analyzed using ProtParam (https://web.expasy.org/protparam/). The secondary structure and 3-D structure of HpaXpm were predicted using the I-TASSER server (https://zhanglab.ccmb.med.umich.edu/I-TASSER/). For the multiple alignment of the HpaXpm amino acid sequence and of other Hpa1 of *Xanthomonas,* CLUSTALW was used. The *Xanthomonas* Hpa1 group includes HpaG-Xag (*X. axonopodis* pv. *glycines*), HpaG-Xcm (*X. citri* pv. *mangiferaeindicae*), Hpa1-Xac (*X. axonopodis* pv. *citri*), Hpa1-Xm (*X. citri* subsp. *malvacearum*), Hpa1-Xoc (*X. oryzae* pv. *oryzicola*), Hpa1Xoo (*X. oryzae* pv. *oryzae*), and XopA-Xcv (*X. campestris* pv. *vesicatoria*). A phylogenetic tree of the complete amino acid sequences of HpaXpm and of the other seven *Xanthomonas* Hpa1 proteins was constructed using the maximum likelihood method (Jones-Taylor-Thornton model) and using the MEGA 6.0 program. The Bootstrap value was set to 1000.

### HpaXpm-treated plant reaction assay

Seeds of tobacco (*Nicotiana tabacum* cv. Samsun-NN) and *Arabidopsis thaliana*, ecotype Columbia were stored in the laboratory at 4 °C. The infected *Tobacco mosaic virus* (TMV) leaves were stored in the laboratory at − 80 °C after rapid freezing in liquid nitrogen. The purified HpaXpm, which was prepared according to the method described above, was diluted to 10 μM in PBS and stored in the laboratory at − 80 °C for use in subsequent experiments. In order to test the heat resistance, HpaXpm was heated at 100 °C for 10 min. Hpa1Xoo protein, which was stored in our laboratory at − 80 °C, was also diluted to 10 μM in PBS and acted as a control.

Assays were performed to determine the activity of unheated HpaXpm and of HpaXpm heated at 100 °C (B-HpaXpm), 150 °C (B-HpaXpm150), or 200 °C (B-HpaXpm200) in terms of eliciting a HR and inducing resistance in tobacco using the methods previously described [3, 10, 33]. The HR assay was performed by injecting HpaXpm (10 μM) and heated HpaXpm (10 μM) into the leaves of 30-day-old seedlings [34]; PBS was used as a negative control and Hpa1Xoo (10 μM) was used as a positive control. Fifteen leaves were used for each treatment with 5 technical replicates and 3 biological replicates. Five days (d) post injection, the leaves were assessed by scoring the HR. The activity level of harpin in terms of eliciting the HR was assessed by determining the ratio of the lesion area to the injected area. The lesion area and the injected area were measured using ImageJ software. According to a previously described method [33], the induced resistance assay was performed by foliar spraying with HpaXpm (10 μM) or B-HpaXpm (10 μM) 12 h before TMV infection; PBS was sprayed as a negative control and Hpa1Xoo was sprayed as a positive control. Fifteen leaves were used for each treatment with 5 technical replicates and 3 biological replicates. The protein-induced defense response was assessed by determining the ratio of necrotic area to total leaf area. The area was measured using ImageJ software 5 d after the inoculations.

To explore the effect of HpaXpm and B-HpaXpm on plant growth, the root length of plants grown from protein-treated seeds was measured. PBS-treated seeds acted as a negative control; Hpa1Xoo-treated seeds acted as a positive control. Seeds of *A. thaliana* were soaked in a diluted sodium hypochlorite solution (1.5% (w/v)) for 10 min, followed by washing with ultrapure water at least three times and then chilled in ultrapure water at 4 °C for 4 d as previously described [3]. Next, the seeds were soaked in 15 μg ml^− 1^ of HpaXpm, B-HpaXpm, Hpa1Xoo, or PBS solutions for 6 h before placing the seeds on 10 cm^2^ plates containing Murashige and Skoog (MS) medium [24]. Thirty seeds were used for each treatment with 10 technical replicates and 3 biological replicates. The seeded plates were placed vertically in 24 °C chambers with a 14-h day: 10-h night cycle. Root lengths and fresh weight were measured at 10 days post treatment (dpt).

### qRT-PCR assay

After treating fully expanded tobacco leaves with either the prepared proteins (10 μM HpaXpm, B-HpaXpm, B-HpaXpm150, or B-HpaXpm200) or PBS (as control), quantitative real-time PCR (qRT-PCR) was performed to measure the relative transcription expression of the HR marker genes *Hsr203J* [35] and *Hin1* [36], the defense-related gene *NPR1* [37], and the plant growth enhancement (PGE)-related gene *NtEXP6* [38]. The *EF-1a* gene [3], which is highly conserved and constitutively expressed in tobacco, was used for normalization of qRT-PCR in tobacco. The expression levels of these marker genes were recorded at 1, 3, and 6 hpt, and expression levels in plants treated with PBS were used as controls. Nine leaves were used for each treatment with 3 technical replicates and 3 biological replicates. RNA isolation [39, 40] and the qRT-PCR assay [3, 40] were performed as described previously. Data were normalized to the *EF-1a* gene using 2^-ΔΔCT^. The sequences of qRT-PCR primers used in this study were as follows: Hsr203J-F 5′-AGCTATGAAAAAGGGGGAAA-3′, Hsr203J-R 5′-AACCATTAGAACGTGACAATC-3′; Hin1-F 5′-TGACTATTAGAAACCCCAACA-3′, Hin1-R 5′-CTTCCATCTCATAAACCCCT-3′; NPR1-F 5′-TTCGTCGCTACCGATAACAC-3′, NPR1-R 5′-TTCTCGCTGACAAAACGCAC-3′; NtEXP6-F 5′- CTCAATGGTGTCATGCTGGA-3′, NtEXP6-R 5′-GCCGCTTCAGCTCTTCTACA-3′; EF-1a-F 5′- ATCAATCCAGGTCATCATCA-3′, EF-1a-R 5′- AAGTTCCTTACCAGAACGCC-3′.

### Statistical analysis

All experiments were carried out three biological replicates. Quantitative data were analyzed with the Statistical Program for Social Science 17.0 software. A one-way analysis of variance (ANOVA) followed by Bonferroni post hoc test (*p* < 0.05) was performed to determine significant differences between treatments.

### Accession number

The GenBank accession number of the *HpaXpm* gene described in this study is KY765410.1 (https://www.ncbi.nlm.nih.gov/nuccore/KY765410.1).

## Results

### HpaXpm identification, characteristics, structure prediction, and phylogenetic relationships among Hpa1

**Fig. 1:**
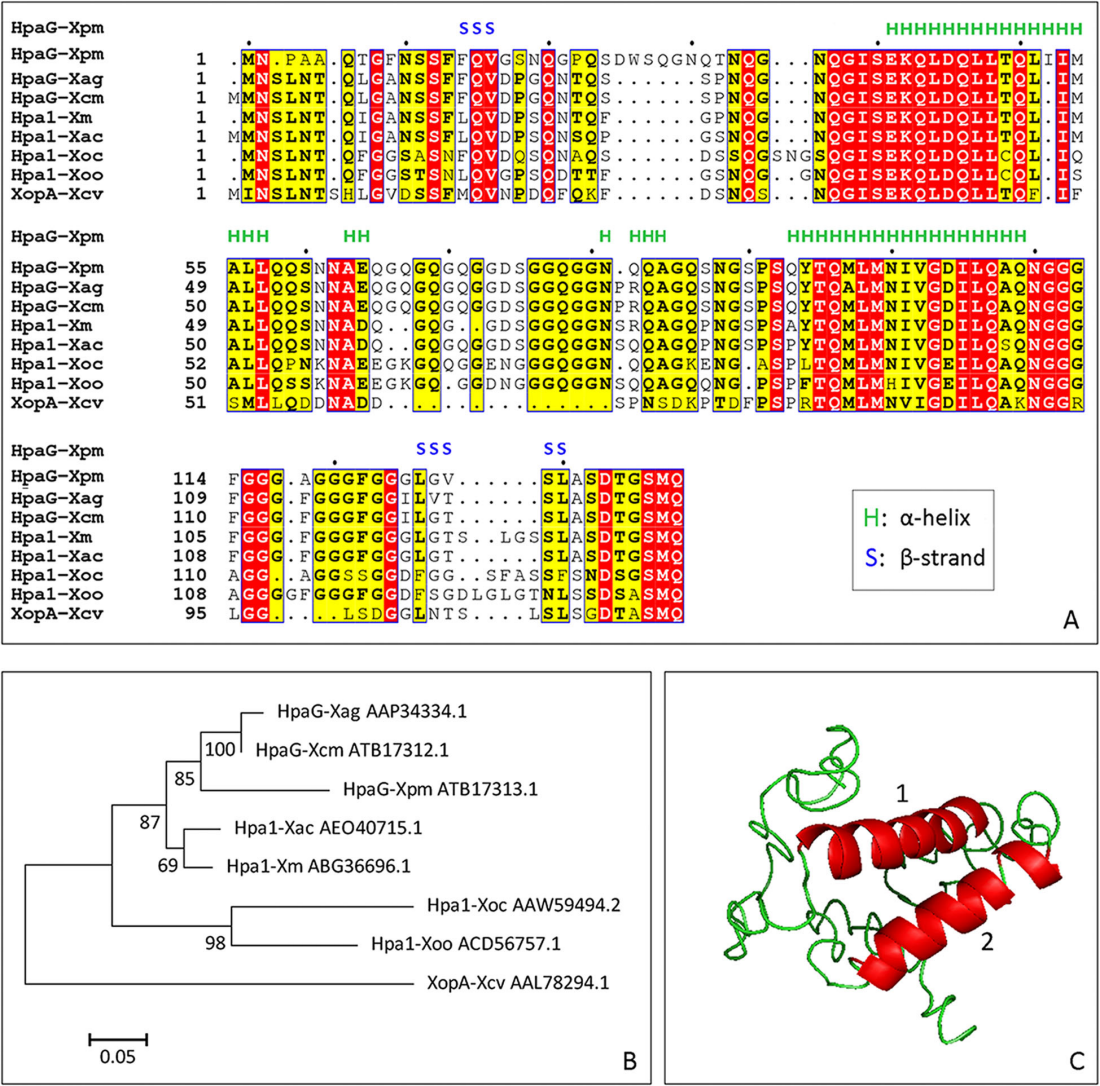
Multiple alignment of the sequence, phylogenetic tree, and prediction of HpaXpm structure. **a** Multiple sequence alignment of HpaXpm with other members of the *Xanthomonas* Hpa1 group, and the secondary structure of HpaXpm. For the multiple alignment of the amino acid sequence of HpaXpm and of other Hpa1 of *Xanthomonas*, CLUSTALW was used. The secondary structure of HpaXpm was predicted using the I-TASSER server. **b** A maximum likelihood bootstrap tree derived from the amino acid sequences of *Xanthomonas* Hpa1 proteins using the MEGA 6.0 program. Protein accession numbers are indicated after each harpin. Harpin abbreviations: Xag, *X. axonopodis* pv. *glycines*; Xcm, *X. citri* pv. *mangiferaeindicae*; Xpm, *X. phaseoli* pv. *manihotis*; Xac, *Xanthomonas axonopodis* pv. *citri*; Xm, *X. citri* subsp. *malvacearum*; Xoc, *X. oryzae* pv. *oryzicola*; Xoo, *X. oryzae* pv. *oryzae*; Xcv, *X. campestris* pv. *vesicatoria*. **c** Ribbon representation of the 3-D structure of HpaXpm using the I-TASSER server. Residues involved in the α-helix are highlighted in red. Stretch-1: 41–64 EKQLDQLLTQLIIMALLQQSNNAE. Stretch-2: 81–109 NQQAGQSNGSPSQYTQMLMNIVGDILQAQ

**Fig. 2:**
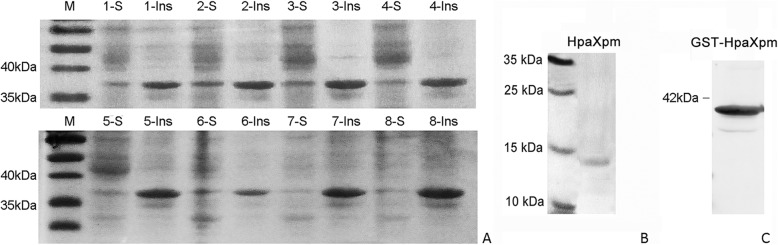
SDS-PAGE and western blot of GST-HpaXpm expression. **a** SDS-PAGE analysis of crude protein. **b** SDS-PAGE analysis of purified HpaXpm protein. **c**, Western blot of GST-HpaXpm. In A, lane M indicates the protein marker; lanes 1–8 indicate the soluble and insoluble proteins of GST-*HpaXpm* gene expression under different inducing conditions: 1, 2, 3, and 7 induced by 0.05 mM IPTG; 4, 5, 6, and 7 induced by 0.1 mM IPTG; 1, 4, 6, and 7 induced at 37 °C; 2, 3, 4, and 8 induced at 28 °C; 1, 3, 4, and 5 induced at 3 h; 2, 6, 7, and 8 induced at 5 h. S, soluble protein. Ins, insoluble protein. Each protein preparation (20 μl) was loaded in the gel. In B, SDS-PAGE assays of purified HpaXpm proteins (theoretical size: 13.8 kDa). In C, the western blot shows the soluble protein of GST-HpaXpm (theoretical size: 38.8 kDa) after induction for 5 h at 37 °C in 0.05 mM IPTG. GST-HpaXpm was immunoblotted with an anti-GST with antibody

**Fig. 3:**
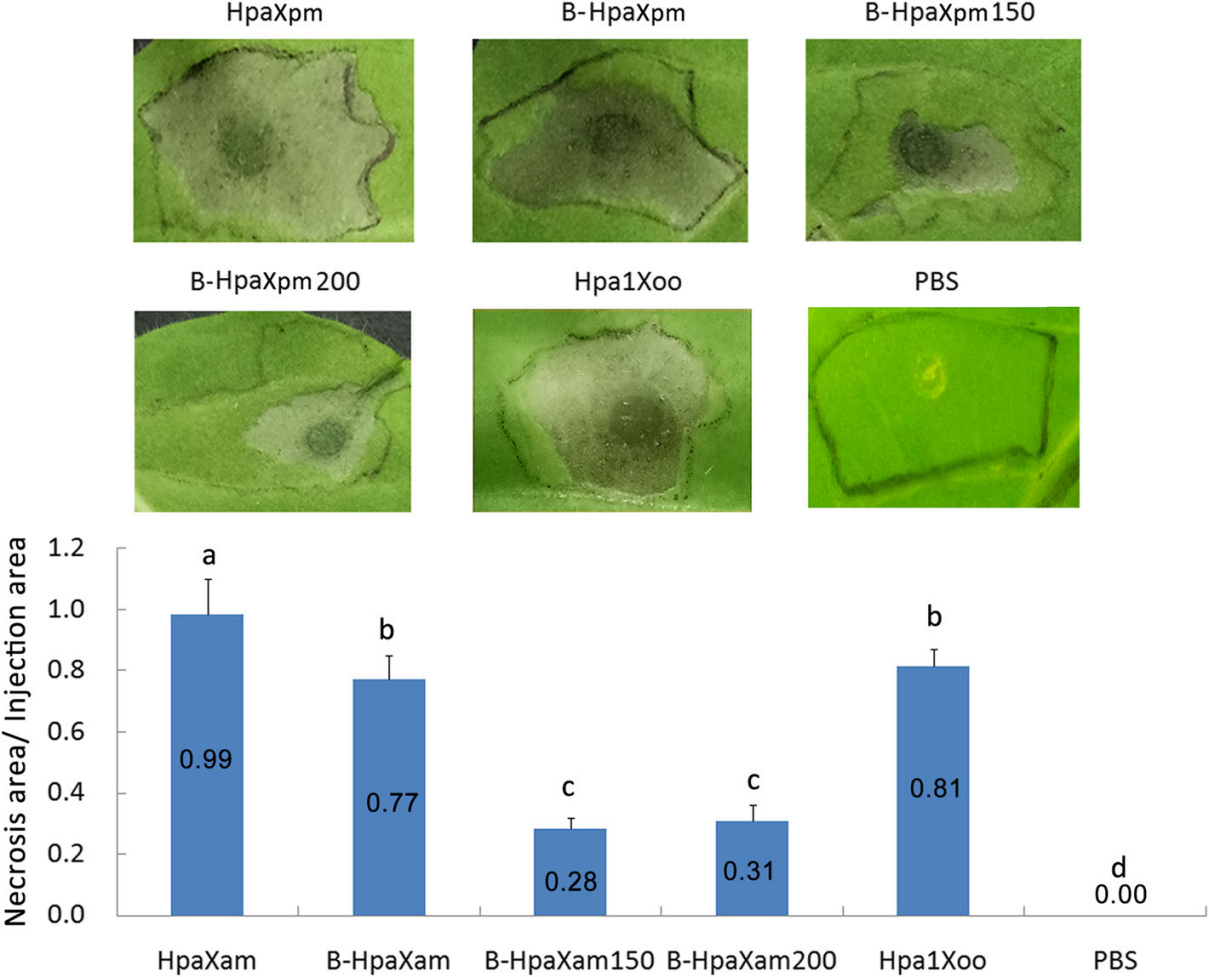
Comparison of hypersensitive response elicitor activity in tobacco leaves 5 days after infiltration. Tobacco leaves were infiltrated with HpaXpm, B-HpaXpm, B-HpaXpm150, or B-HpaXpm200, with PBS as a negative control and Hpa1Xoo as a positive control. The injection range is indicated by a dashed line. Tobacco hypersensitive cell death levels were determined by comparing the ratio of the lesion area to the injected area. Quantitative data are given as mean values ± statistical estimates of standard error of mean (SEM). Different lowercase letters above the bar graphs indicate significant differences in multiple comparisons of data from the different proteins; *P* < 0.05; *n* = 15 leaves from 3 independent experiments each involving 5 leaves

The NCBI blast showed that the nucleotide sequence of *hpa1* was similar to that of the *Xpm* nucleotide sequence at position 488,218–488,631, with identities of 88.04% (data not shown). Therefore, the sequence at position 488,218–488,631 of the *Xpm* genome sequence was initially determined to be HpaXpm. To further confirm that HpaXpm is a harpin protein, we performed bioinformatic analyses of the amino acid sequence of HpaXpm. ProtParam analysis revealed that HpaXpm is acidic (theoretical pI: 3.57), rich in glycine (23.2% of the total amino acids) but lacks cysteine (0% of the total amino acids) (Fig. 1a). The homology alignment of the primary sequence indicated that HpaXpm was most similar to HpaG-Xcm and HpaG-Xag, with a sequence similarity of 87.8 and 86.3%, respectively (Fig. 2a). The phylogenetic tree generated using the maximum likelihood method grouped harpins in the Hpa1 group into three clades, with HpaG-Xag, HpaG-Xcm, HpaXpm, Hpa1-Xac, and Hpa1Xm in clade I, Hpa1-Xoc and Hpa1Xoo in clade II, and XopA-Xcv in clade III (Fig. 1b). The predicted secondary structure (Fig. 1a) and 3-D structure (Fig. 1c) indicated that HpaXpm protein has two β-strand domains and two major α-helical domains located at the N- and C-terminal regions, respectively. In summary, HpaXpm was identified as a novel harpin-like protein. The nucleotide and amino acid sequences of HpaXpm were submitted to the NCBI GenBank under accession number KY765410.1. Furthermore, HpaXpm belongs to the same subgroup of the Hpa1 group as HpaG-Xag and HpaG-Xcm.

### HpaXpm expression and western blot analysis

In order to overproduce HpaXpm, we induced HpaXpm expression under different conditions. SDS-PAGE analysis confirmed differences in the expression efficiency of pGEX-GSTHpaXpm from induced BL21/pGEX-GSTHpaXpm under different induction conditions (Fig. 2a). The GST-HpaXpm was effectively induced by 0.05 mM IPTG at 28 °C for 5 h. The GST tag was cleaved from the GST-HpaXpm fusion protein to obtain the purified HpaXpm (theoretical size: 13.8 kDa), which was approximately 13.8 kDa in size (Fig. 2b). Western blot analysis confirmed the efficiency of the GST-HpaXpm (theoretical size: 38.8 kDa). The approximately 38.8 kDa band detected by a rabbit monoclonal antibody GST was the GST-HpaXpm (Fig. 2c).

### HR induced by HpaXpm

In order to compare the intensity of the HR stimulated by HpaXpm, B-HpaXpm, B-HpaXpm150, B-HpaXpm200, Hpa1Xoo and PBS treatments, the HpaXpm, B-HpaXpm, B-HpaXpm150, B-HpaXpm200, Hpa1Xoo, and PBS activity levels were assessed by measuring the ratio of the necrotic area to the injected area of leaves treated with each solution. HpaXpm, B-HpaXpm, B-HpaXpm150, B-HpaXpm200, and Hpa1Xoo induced HR; PBS did not induce HR (Fig. 3). The ratio of the lesion area to the injected area induced by HpaXpm (ratio value: 0.99) was significantly higher (*P* < 0.05) than that induced by Hpa1Xoo (ratio value: 0.81) or PBS (ratio value: 0.00); the ratio of the lesion area to the injected area induced by Hpa1Xoo was significantly higher (*P* < 0.05) than that induced by PBS (Fig. 3). The ratio of the lesion area to the injected area induced by HpaXpm was significantly higher (*P* < 0.05) than that induced by B-HpaXpm (ratio value: 0.77), B-HpaXpm150 (ratio value: 0.28), or B-HpaXpm200 (ratio value: 0.31); the ratio of the lesion area to the injected area induced by B-HpaXpm was significantly higher (*P* < 0.05) than that induced by B-HpaXpm150 or B-HpaXpm200; however, the difference between B-HpaXpm150 and B-HpaXpm200 was not significant (*P* > 0.05). These results indicated that HpaXpm induced a stronger HR than Hpa1Xoo in non-host tobacco. In addition, after HpaXpm was subjected to high temperature treatments of 100 °C, 150 °C, or 200 °C, HpaXpm still showed activity comparable to that of non-heat-treated HpaXpm.

**Fig. 4:**
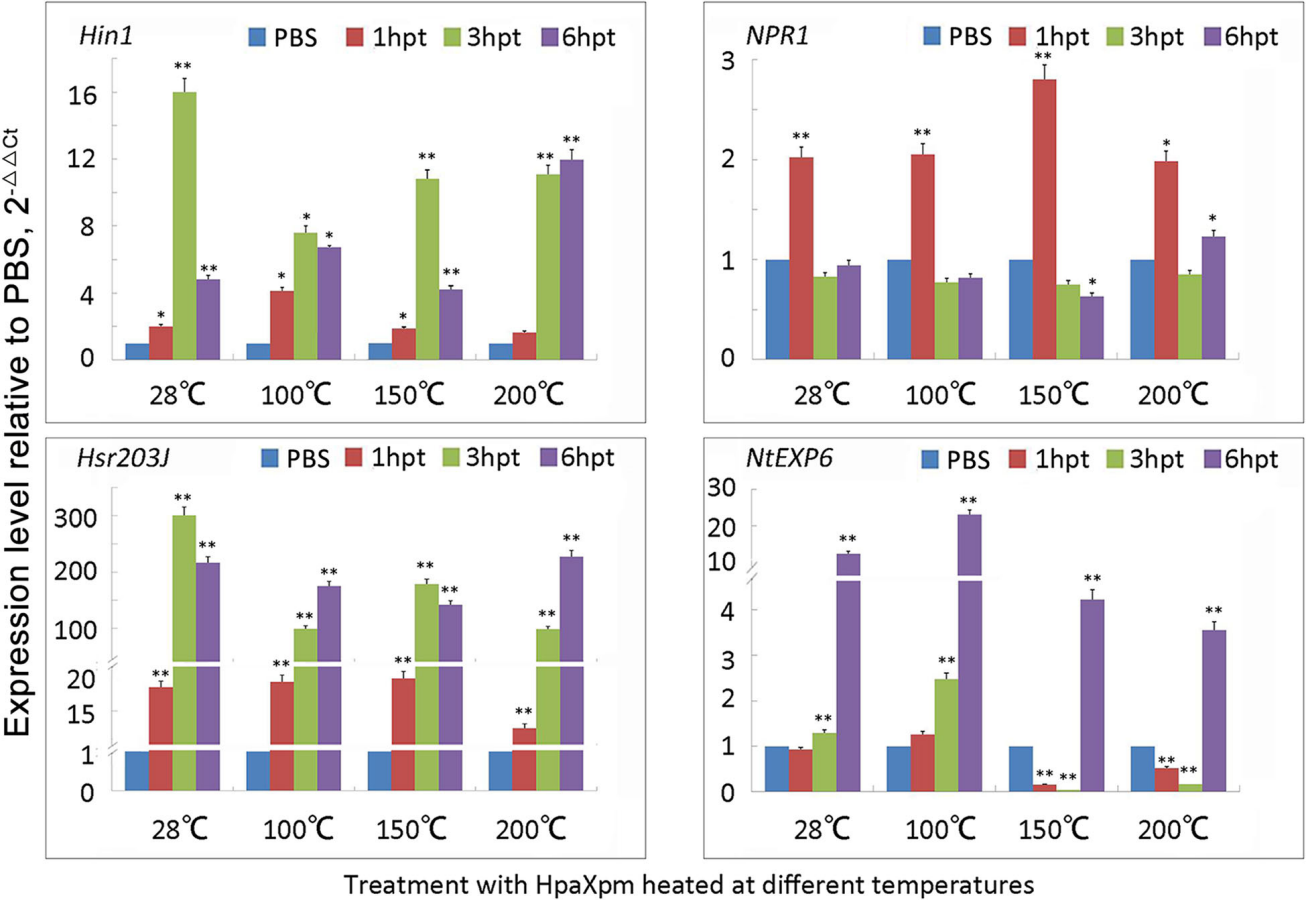
Real-time quantitative PCR (qRT-PCR) analyses of HR markers, defense markers, and PGE markers. qRT-PCR analysis of the transcript levels of the HR-related genes *Hin1* and *Hsr203J*, the defense-related gene *NPR1*, and the PGE-related gene *NtEXP6* was performed on RNA isolated from leaves harvested at 1, 3, and 6 h post treatment (hpt) and subjected to different treatments. A PBS treatment was used as a control. 28 °C, 100 °C, 150 °C, and 200 °C indicate HpaXpm, HpaXpm heated at 100 °C, HpaXpm heated at 150 °C, and HpaXpm heated at 200 °C, respectively. The qRT-PCR data were reported and calculated based on the normalization gene EF-1a using the 2^-ΔΔCT^ method. Expression levels of HR markers, defense markers, and PGE markers in response to HpaXpm treatment relative to expression levels induced by PBS (control) treatment. Quantitative data are given as mean values ± SEM. Asterisks indicate treatments that induced a significant difference (^*^P < 0.05; ^**^P < 0.01) relative to that of the PBS-treated controls; *n* = 9 leaves from 3 independent experiments each involving 3 leaves

To determine the activation of the molecular HR in tobacco plants treated with HpaXpm, B-HpaXpm, B-HpaXpm150, or B-HpaXpm200, we used qRT-PCR to monitor the mRNA accumulation of the HR marker genes *Hin1* and *Hsr203J* relative to that of the PBS-treated controls. Both *Hin1* and *Hsr203J* genes are marker genes for HR, which are expressed specifically in plants undergoing a HR [41, 42]. At 1, 3, and 6 h post treatment (hpt), both *Hin1* and *Hsr203J* genes in leaves treated with HpaXpm were significantly upregulated (*P* < 0.01) relative to *Hin1* and *Hsr203J* expression in PBS-treated leaves (Fig. 4). In addition, the expression of both genes increased with time from 1 hpt with B-HpaXpm, B-HpaXpm150, or B-HpaXpm200. At 3 and 6 hpt, both genes in leaves treated with B-HpaXpm, B-HpaXpm150, or B-HpaXpm200 were significantly upregulated (*P* < 0.01) relative to expression levels in PBS-treated leaves. HpaXpm and the heated HpaXpm protein can effectively induce the expression of *Hin1* and *Hsr203J*.

### Induction of disease resistance by HpaXpm

**Fig. 5:**
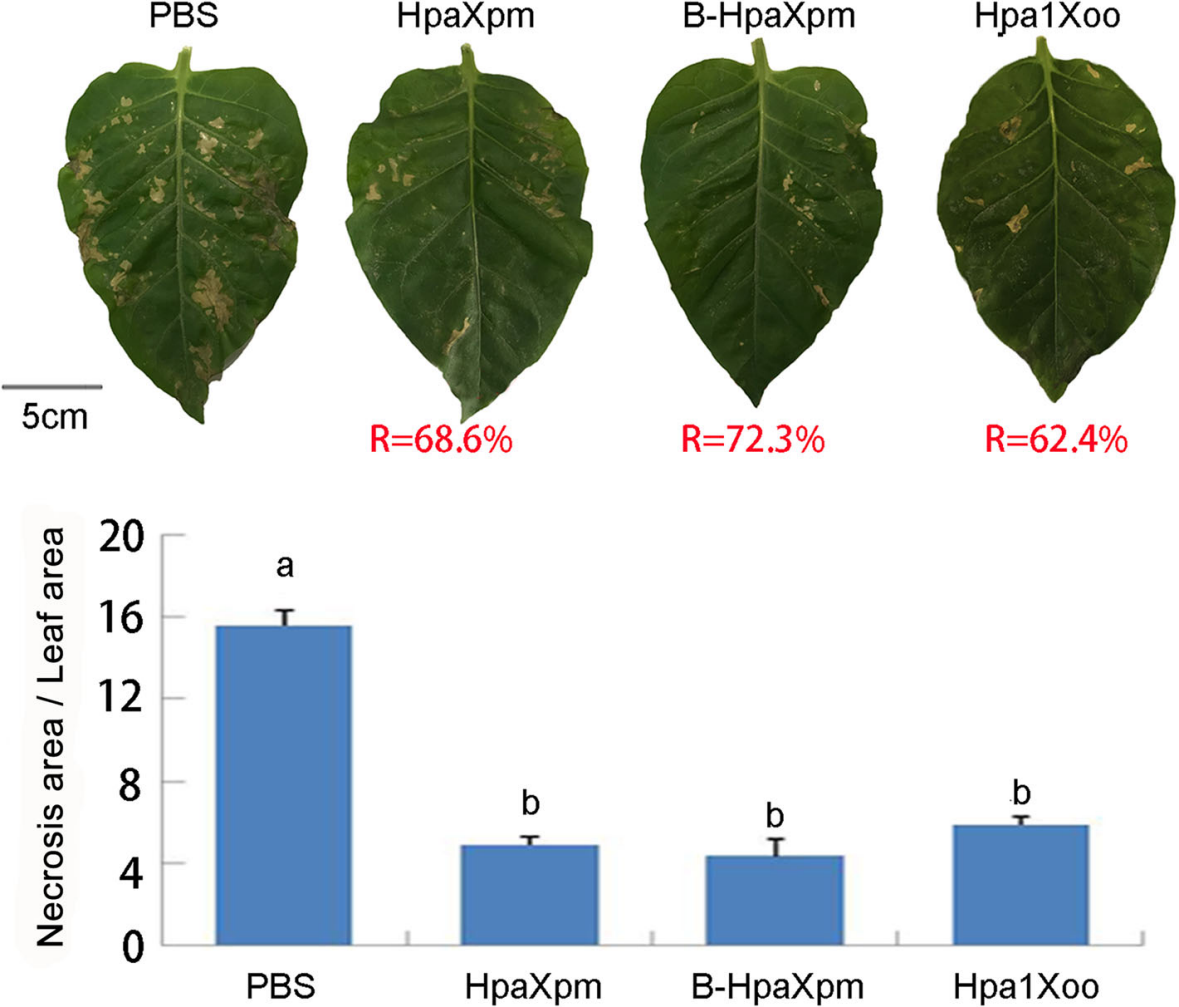
Responses of tobacco leaves to TMV after treatment with HpaXpm or B-HpaXpm. Leaves were inoculated with *Tobacco mosaic virus* (TMV) 12 h after spraying with HpaXpm or B-HpaXpm; leaves sprayed with PBS acted as a negative control and leaves inoculated with Hpa1Xoo acted as a positive control. Treated leaves were photographed 5 days after inoculation. The disease severity of leaves was determined by comparing the ratio of the lesion area to the whole leaf area. Quantitative data are given as mean values ± SEM. Different lowercase letters above the bar graphs indicate significant differences in multiple comparisons of data from the different proteins; *P* < 0.05; n = 15 leaves from 3 independent experiments each involving 5 leaves. The mean reduction in disease severity of leaves treated with HpaXpm, B-HpaXpm, or Hpa1Xoo, relative to those treated with PBS, is shown in red

In order to compare the intensity of the defense response induced in non-host plants by HpaXpm, B-HpaXpm, Hpa1Xoo, and PBS treatments, the activity levels of HpaXpm, B-HpaXpm, Hpa1Xoo, and PBS were assessed by measuring the ratio of the area of necrosis produced by TMV in leaves treated with each solution to the total leaf area. After 5 d, fewer lesions were observed in plants treated with HpaXpm, B-HpaXpm, or Hpa1Xoo than in negative control plants treated with PBS (Fig. 5). Statistical analysis of the necrotic area and the leaf area using ImageJ software showed that the necrotic area of leaves treated with HpaXpm, B-HpaXpm, or Hpa1Xoo was 68.6, 72.3%, or 62.4% smaller, respectively, than that of PBS-treated leaves (*P* < 0.05), suggesting that these leaves were less susceptible to TMV infection. These results indicate that HpaXpm and B-HpaXpm are capable of inducing disease resistance in tobacco even more strongly than Hpa1Xoo.

To determine the expression level of the defense-related gene *NPR1* in tobacco leaves treated with HpaXpm, B-HpaXpm, B-HpaXpm150, B-HpaXpm200, or PBS, qRT-PCR analyses were conducted using *EF1a* as a standard. The *NPR1* gene was significantly upregulated (*P* < 0.05) at 1 hpt by all the harpin treatments relative to *NPR1* expression in PBS-treated leaves (Fig. 4). Interestingly, the highest levels of *NPR1* expression were observed in leaves treated with B-HpaXpm150. These results indicate that both unheated HpaXpm and heated HpaXpm can effectively induce *NPR1* expression.

### Plant growth promotion by HpaXpm

**Fig. 6:**
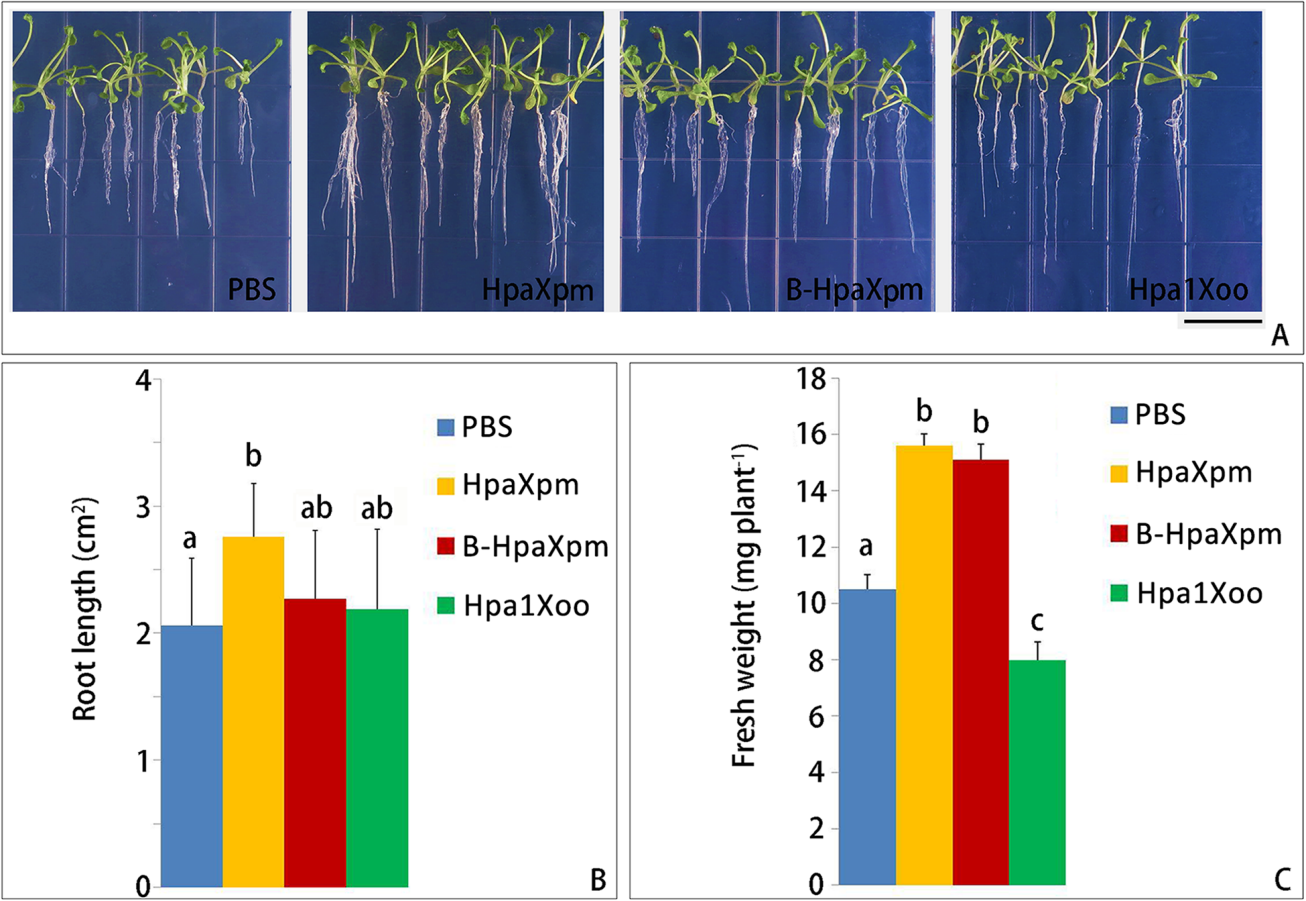
Effects of HpaXpm and B-HpaXpm on the growth of *Arabidopsis*. **a** Appearance of *Arabidopsis* roots grown on MS medium. Root growth represents the growth-promoting effect of each 10 μM protein solution on the seeds. **b** and **c** Quantification of root growth and fresh weight on MS medium at 10 dpt. Seeds were soaked in a HpaXpm, B-HpaXpm, Hp1Xoo, or PBS solution for 6 h before placing the seeds on 10 cm^2^ plates containing MS medium. The seeded plates were placed vertically in 24 °C chambers with a 14-h day: 10-h night cycle. Quantitative data are given as mean values ± SEM. Different lowercase letters above the bar graphs indicate significant differences in multiple comparisons of data from the different proteins; *P* < 0.05; *n* = 30 seeds from 3 independent experiments each involving 10 seeds

To explore the activity of HpaXpm in the growth promotion of roots, the root length of plants grown from seeds treated with HpaXpm or B-HpaXpm was measured every 5 d, with PBS- or Hpa1Xoo-treated seeds acting as controls (Fig. 6). At 10 dpt, the roots of plants grown from seeds treated with HpaXpm were significantly longer (*P* < 0.05) than those of plants grown from seeds treated with PBS; the roots of plants grown from seeds treated with B-HpaXpm were longer (*P* > 0.05) than those of plants grown from seeds treated with Hpa1Xoo or PBS; the roots of plants grown from seeds treated with Hpa1Xoo were longer (*P* > 0.05) than those of plants grown from seeds treated with PBS (Fig. 6b). At 10 dpt, the fresh weights of plants grown from seeds treated with HpaXpm or B-HpaXpm were significantly greater (*P* < 0.05) than those of plants grown from seeds treated with PBS or Hpa1Xoo (Fig. 6c). Although the roots of plants grown from seeds treated with Hpa1Xoo were longer than those of plants grown from seeds treated with PBS, the fresh weights of plants grown from seeds treated with Hpa1Xoo were lower than those of plants grown from seeds treated with PBS.

The qRT-PCR analysis revealed that at 6 hpt of leaves with untreated HpaXpm or the heated protein, *NtEXP6* was significantly upregulated (*P* < 0.01) relative to *NtEXP6* expression in PBS-treated leaves (Fig. 4). This suggests that HpaXpm and the heated HpaXpm protein may have promoted growth by enhancing the expression of the *NtEXP6* gene.

## Discussion

To date, previous studies have reported harpins from pathogens of citrus, cotton, rice, soybeans, and peppers [2, 4, 10, 11, 23, 43, 44]. In the present study, we isolated a new class of harpin, HpaXpm, from cassava blight bacteria *Xanthomonas phaseoli* pv. *manihotis*HNHK. Moreover, our investigation of the phylogenetic relationships among harpins in the Hpa1 group revealed three clades, with HpaG-Xag, HpaG-Xcm, HpaXpm, Hpa1-Xac, and Hpa1Xm in clade I, Hpa1-Xoc and Hpa1Xoo in clade II, and XopA-Xcv in clade III. HpaXpm was added to the Hpa1 group and grouped into the same subgroup as HpaG-Xag, HpaG-Xcm, Hpa1-Xac, and Hpa1-Xm. The addition of HpaXpm improves our understanding of the evolutionary relationships of harpins.

In the present study, we heated HpaXpm to 150 °C or 200 °C, and surprisingly found that HpaXpm still has the ability to stimulate tobacco HR, disease resistance, and growth promotion after these high temperature treatments. Previous studies have shown that harpin is heat resistant by heating the protein to 100 °C [3, 10]. However, the maximum temperature that harpin protein can withstand is unknown; whether different temperatures affect the activity of harpin protein in different ways is unclear; and whether the high thermal stability of the harpin protein is due to structural specificity or because the structure has no effect on the thermal stability of harpin is also unknown. The answers to these three questions could lay the foundations for exploring the heat-resistance mechanism of harpin protein. In this study, we observed that HpaXpm heated to 100 °C, 150 °C, or 200 °C still has the ability to induce HR; however, its ability to induce HR was different at different temperatures. In previous studies, the persistence of life at extreme temperatures depends in part on the level of protein adaptability [45]. Furthermore, it is well-known that the function of proteins is closely related to their structure. We hypothesize that the extreme heat resistance of HpaXpm is because the structure of harpin is very stable and, therefore, the HpaXpm structure is less affected by temperature. Therefore, in this study, the secondary and tertiary structures of HpaXpm were predicted. These predictions suggest that HpaXpm proteins have two β-strand domains and two major α-helical domains located at the N- and C-terminal regions, respectively. Furthermore, previous studies [3, 46, 47] have proposed that the heat resistance of harpins may be closely related to their amino acid composition because the cysteine residue content is related to the conversion of two disulfide bonds. According to the hypothesis presented in these previous studies [3, 46, 47], HpaXpm may be heat-resistant due to the absence of cysteine in its primary structure. Investigating the heat resistance mechanism of HpaXpm could help to locate factors that contribute to the unusual stability of harpins at extreme temperatures and also identify new ways of improving the thermal stability of beneficial but heat-sensitive proteins. Therefore, in future studies, we will continue to conduct in-depth investigations of the relationship between the harpin structure and the heat resistance mechanism to verify our hypothesis.

In this study, like other harpins, HpaXpm elicits tobacco HR, stimulates defense responses, and promotes plant growth. All these plant responses were stronger than those elicited by Hpa1Xoo. As previously reported, transgenic technology has been used to transform cotton with the *hpa1Xoo* gene, which confers resistance to multiple pathogens [48], and the expression of Hpa1Xoo in transgenic tobacco induces pathogen defense [37]. An exploration of the diverse functional aspects of HpaXpm could provide possibilities for future applications, such as increasing plant yield or quality. In addition, our results suggest that HpaXpm could be more valuable than Hpa1Xoo in terms of agricultural development and applications. Our next steps will be to conduct a field research study to verify whether HpaXpm has greater potential value than Hpa1Xoo in field applications.

## Conclusions

In this study, we describe HpaXpm, a new member of the harpin family, which is not only stable at high temperatures (up to 200 °C) but also has the ability to stimulate non-host HR, defense responses, and plant growth. In addition, HpaXpm induces stronger plant responses than those elicited by Hpa1Xoo. HpaXpm could be more valuable than Hpa1Xoo in terms of agricultural development and applications.

## Data Availability

All data generated or analyzed during this study are included in this published article.

## Abbreviations

dpt: days post treatment
*Hpa*: *hrp*-associated
hpt: hours post treatment
*Hrc*: *hrp*-conserved
*Hrp*: hypersensitive response and pathogenicity
IPTG: isopropyl-β-D-thiogalactopyranoside
MS: Murashige and Skoog
PBS: phosphate-buffered saline
PGE: plant growth enhancement
qRT-PCR: quantitative real-time PCR
SAR: systemic-acquired resistance
SEM: statistical estimates of standard error of mean
TMV: *Tobacco mosaic virus*
*Xoo*: *Xanthomonas oryzae* pv. *oryzae*
*Xpm*: *Xanthomonas phaseoli* pv. *manihotis*
